# Complete genome sequence of a pESI-Carrying *Salmonella* Infantis from raw chicken meat in a Metro Manila wet market, Philippines

**DOI:** 10.1128/mra.00527-25

**Published:** 2025-07-28

**Authors:** Michael Joseph M. Nagpala, Andrew D. Montecillo, Jonah Feliza B. Mora, Rance Derrick N. Pavon, Homer D. Pantua, Windell L. Rivera

**Affiliations:** 1 Pathogen-Host-Environment Interactions Research Laboratory, Institute of Biology, College of Science, University of the Philippines Diliman 172582, Quezon City, Philippines; 2 Microbiology Division, Institute of Biological Sciences, College of Arts and Sciences, University of the Philippines 482308, Los Baños, Philippines; 3 BioAssets Corporation, City of Santo Tomas, Batangas, Philippines; Queens College Department of Biology, Queens, New York, USA

**Keywords:** *Salmonella* Infantis, pESI plasmid, antimicrobial resistance, complete genome

## Abstract

We report the complete genome sequence (4,727,598 bp chromosome, 308,757 bp plasmid of emerging *S*. Infantis (pESI), and 5,423 bp pSCD6R1a_5.4k plasmid) of *Salmonella enterica* serovar Infantis SCD6R1a, isolated from raw chicken meat sold at a wet market in Metro Manila, Philippines. The isolate harbored a mobilizable pESI plasmid, computationally predicted to carry multiple virulence factors and antimicrobial resistance genes.

## ANNOUNCEMENT

*Salmonella enterica* serovar Infantis has emerged as a predominant serovar in chicken meat across the United States, Europe, and Asia (1), largely attributed to the “plasmid of emerging *S*. Infantis” or pESI, which carries multiple antimicrobial resistance genes (ARGs) and virulence factors that enhance its fitness and spread (2). This study presents the first complete genome of a pESI-carrying *S*. Infantis isolate from the Philippines, providing a valuable reference for understanding the plasmid’s global spread and its role in the rising dominance of *S*. Infantis in poultry.

The chicken sample was collected from a public market in San Juan City, Metro Manila (14.605437060675548, 121.02305370000003), following local legislation and institutional requirements. The isolation, DNA extraction, and short-read sequencing of *S. enterica* were previously described (3). For both sequencing workflows, the isolate was cultured in Tryptic Soy Broth at 37°C for 24 h prior to DNA extraction. Long-read sequencing DNA was extracted using IndiSpin Pathogen Kit (Indical Bioscience GmbH, Leipzig, Germany), and sequencing library was prepared with Rapid Barcoding Kit (SQK-RBK114; Oxford Nanopore Technologies, England, UK). Sequencing was performed at the Core Sequencing Laboratory of the BioAssets Corporation using a MinION SpotON R10.4.1. FLO-MIN114 flow cell (ONT) and MinKNOW Software (v24.11.10; ONT). Reads were basecalled with the super accurate model (r1041_e82_400bps_sup_v5.0.0), and adapters were trimmed using MinKNOW’s default parameters.

Reads were filtered using Chopper v0.10.0b (4) with minimum quality and length of 10 and 1,000, respectively. Assembly was carried out using Trycycler v0.5.5 (5), with filtered reads subsampled into 12 subsets targeting a 5 Mbp genome using “Trycycler subsample.” Eight independent read subsets were assembled with either Flye v2.9.5 (6), Miniasm v0.3-r179 (7) and Minipolish v0.1.3 (8), or Raven v1.8.3 (9). The contigs were clustered and assigned read depths using “Trycycler cluster,” then circularized using “Trycycler reconcile.” Multiple sequence alignments were generated using MUSCLE via “Trycycler msa,” and the original filtered reads were assigned to clusters using “Trycycler partition” (10). Consensus sequences for each cluster were then generated using “Trycycler consensus” and merged into a final consensus assembly. The assembly was polished using Medaka v2.0.1 (https://github.com/nanoporetech/medaka) and further refined with Illumina reads (SRR33422847) using Polypolish v0.6.0 (11). A 5,423 bp plasmid was identified using Unicycler v0.5.1 (12) and was merged into the polished assembly. Annotation was performed using the NCBI Prokaryotic Genome Annotation Pipeline, and serotyping was conducted using SeqSero2 v1.3.1 (13). Default parameters were applied unless stated otherwise.

A total of 1,179,078 short-read sequences (351,179,886 bp) and 157,003 long-read sequences (644,027,413 bp; N50 = 5,752 bp; mean quality = 19.6) were used to assemble the *S*. Infantis genome. The final assembly yielded a closed, circular 4,727,598 bp chromosome (52.3% GC), a 308,757 bp pESI (50.49% GC), and a 5,423 bp plasmid (47.15% GC), containing 4,931 genes, including 4,630 coding sequences. Coverage was 67.7X (chromosome), 69.5X (pESI), and 51.6X (small plasmid) for short reads; and 138.4X (chromosome), 95.0X (pESI) for long reads. The pESI plasmid carried multiple ARGs (Table 1) and pESI-specific genes (14).

**TABLE 1:** SCD6R1a pESI plasmid encoding multiple ARGs^*a*^

Antimicrobial resistance genes and annotation	Gene start position (nucleotides)	Gene end position (nucleotides)
*aadA1* — ANT(3″)-Ia family aminoglycoside nucleotidyltransferase	98,776	99,681
*qacEdelta1* — quaternary ammonium compound efflux SMR transporter	99,845	100,192
*sul1* — sulfonamide-resistant dihydropteroate synthase	100,186	101,025
*tet(A*) — tetracycline efflux MFS transporter	113,542	114,741
*bla_CTX-M-65_* — extended-spectrum class A beta-lactamase	215,591	216,466
*floR* — chloramphenicol/florfenicol efflux MFS transporter	222,281	223,495
*aph (4)-Ia* — aminoglycoside O-phosphotransferase	230,093	231,118
*aac (3)-IVa* — aminoglycoside N-acetyltransferase	231,347	232,123

^*a*^ The genome was analyzed using NCBI Prokaryotic Genome Annotation Pipeline. The computationally predicted gene names and positions are displayed below.

## Data Availability

The complete genome sequence has been deposited in GenBank under accession nos. CP190247, CP190248, and CP190249. The raw sequencing data have been deposited in the NCBI Sequence Read Archive under accession nos. SRR33422847 (short reads) and SRR33422846 (long reads). The genome is associated with BioSample accession number SAMN44339505.
